# Surgical approach in a patient with Multiple Symmetrical Lipomatosis: Case Report

**DOI:** 10.1590/S1808-86942012000100024

**Published:** 2015-10-20

**Authors:** Francisco Sales de Almeida, Paulo Roberto Pialarissi, Samuel Lima Silva, Leandro Henrique de Oliveira Almeida, Thiago de Almeida Reis

**Affiliations:** a Doctoral degree, USP/SP, Staff of the Odontomed Hospital.; b Doctoral degree, USP/SP, Full professor at the PUC/SP.; c Medical undergraduate student in the Itajuba Medical School, MG.; d Medical undergraduate student in the Juiz de Fora Medical School, MG.; e Medical undergraduate student in the Souza Marques Medical School, Rio de Janeiro, RJ.

**Keywords:** classification, diagnosis, lipomatosis, multiple symmetrical, surgery, plastic

## INTRODUCTION

Multiple symmetric lipomatosis (MSL) may affect large portions of the body and cause deformity; it may also present atypically. The esthetic aspect may be compromised depending on the extent of the tumor; anatomical, psychological, psychosomatic, and physiological aspects may also be affected.

In 1888 Otto Wilhelm Madelung, a surgeon, published a study of 33 patients with symmetric fat tumors that involved the neck and shoulders; around the neck it was distributed as a “horse's collar”^1^.

MSL affects mostly white people; the fat deposits arise after 20 years of age. The incidence is 1: 25,000, and the male to female ratio is 15:1 to 30:1^2^.

The purpose of this study was to present the treatment of MSL and to describe the steps of surgery. The justification for this case report is that there has been little consensus about the treatment, and that there is a paucity of published cases requiring multiple procedures.

## CASE REPORT

MSF, the patient, presented with a tumor that involved the entire neck. Although the tumor caused a significant esthetic deformity, there were no signs or symptoms that suggested involvement of blood vessels, nerves, the digestive or the respiratory systems.

The common anatomical elements of the neck could not be palpated because of the deformity caused by the tumor.

**Figure 1:**
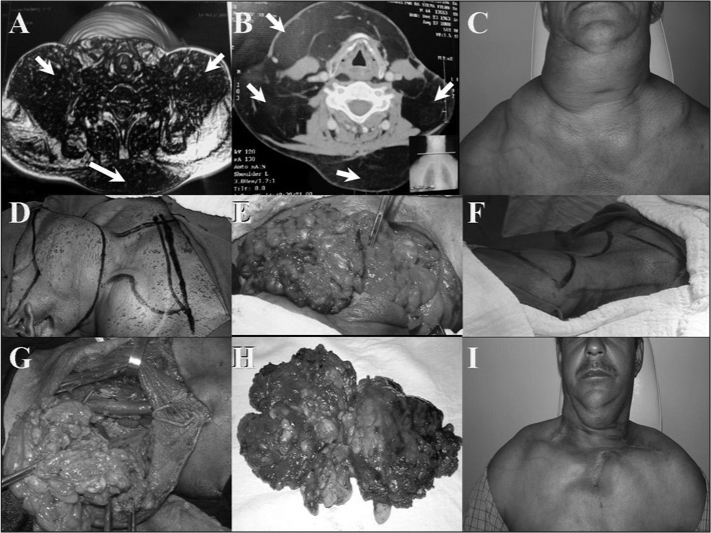
A and B – Computed tomography (axial section) showing a tumor in the neck; C – Preoperative presentation of the neck tumor; D – Marking the surgical approach to the right; E – Exposure of the tumor after resection of skin; F – Marking the surgical approach to the left; G – Exposure of the tumor after resection of skin; H – Surgical specimen; I – Postoperative presentation.

The patient consumed about 1,200 ml of alcohol per day and smoked 25 cigarettes a day. In the family history, the patient had a daughter with a pseudotumor that affects the ocular globes. Computed tomography was done to characterize the tumor. A preoperative assessment consisted of clinical and laboratory tests, which were within normal limits; thus, surgery was indicated (Figure 1 - A, B, and C).

A stepped resection was suggested because of the size of the tumor. The first step consisted of a lateral and posterior resection on the right side of the neck from the midline to the anterior portion of the trapezium muscle. The entire tumor in this side of the neck, including that in the right supraclavicular fossa, was removed (Figure 1 – D and E). Closure was done in two planes, a tube was placed, and compressive dressings were placed over the surgical wound.

A second procedure was done on the left; the same type of approach and procedure was carried out. In both cases, structures such as blood vessels, muscles, nerves, airways, and digestive structures were preserved (Figure 1 – F, G, H, and I).

## DISCUSSION

MSL may present as symmetrical or non-symmetric fat tumors involving the neck and shoulders; it assumes the shape of a “horse's collar”. The patient presented this typical symmetrical aspect; the tumor also extended partially to the thorax^1^.

The patient is brown-skinned and was aged 53 years. The skin color and age agree with published reports. Among many other authors, Meyer states that mostly white individuals are involved, and that the fat appears at the beginning of adulthood^2^.

The pathogenesis of this disease is unknown. This patient consumed alcoholic beverages; reports in the literature have shown a relationship between the action of alcohol on beta-adrenergic receptors and the anti-lipolytic and lipogenic actions of MSL. Other hypotheses have also been raised, such as a possible embryonic origin of this disease; there is a similarity between adipocytes in lipomas and those in brown fat. Another hypothesis suggests that abnormal mitochondrial DNA could cause this disease. A further possibility is differentiation of preadipocytes into mature adipocytes that could point to a different site for lipomas^1^, ^2^.

The treatment of choice is liposuction and open lipectomy. A few authors have argued that diet therapy, metabolic control, use of salbutamol or albuterol, cessation of alcohol, thyroidectomy, and use of vitamins do not yield satisfactory results^3^.

Other authors have suggested low-fat diets, cessation of alcohol use, and physical activities because of recurrences of MSL^4^.

The patient underwent surgery because of the esthetic issues and difficulties in moving the neck and thorax. Staged surgery was done because of the extent of the tumor. Surgeons carrying out this procedure should be experienced; in this case, a sculpturing technique to remove as much of the fat as possible while preserving the normal body contour was done^5^.

## FINAL COMMENTS

The treatment of choice for MSL is open lipectomy. Satisfactory esthetic and functional results are attained with this technique. If necessary, staged surgery is recommended as a feasible and safe approach for cases of large tumors.
